# Assessing the knowledge of emergency medical care personnel in the Free State, South Africa, on aspects of paediatric pre-hospital emergency care

**DOI:** 10.11604/pamj.2019.32.98.17718

**Published:** 2019-03-01

**Authors:** Markes Wayne Butler, Anthonio Oladele Adefuye

**Affiliations:** 1 Free State College of Emergency Care, Free State Department of Health, South Africa; 2 Provincial Disaster Management Centre, Department of Cooperative Governance and Transitional Affairs, Free State Province, South Africa; 3 Division Health Sciences Education, Office of the Dean, Faculty of Health Sciences, University of the Free State, South Africa

**Keywords:** EMC personnel, Free State DoH, paediatrics

## Abstract

**Introduction:**

In South Africa in 2016, injuries accounted for 4 483 deaths of children aged 0-4 years. Prior studies have reported that, in some parts of the country, poor pre-hospital clinical care is a key contributor to the morbidity and mortality of critically ill and injured children. A key component of a coordinated emergency health care system are emergency medical care (EMC) personnel. Here, we assess the knowledge of EMC personnel employed by the Free State Department of Health on aspects of paediatric pre-hospital emergency care.

**Methods:**

This descriptive study used a questionnaire survey to obtain data on the knowledge of Free State EMC personnel on aspects of paediatric pre-hospital emergency care.

**Results:**

Only 197 of the initial 250 questionnaires distributed were returned, giving a response rate of 78.8%. More than half (51.2%) of the participants across the five districts had inadequate knowledge of paediatric pre-hospital emergency care. The majority of EMC personnel could not calculate the paediatric blood pressure for age and did not know the paediatric Glasgow Coma Scale (74.0% and 53.4% respectively; P < 0.0001 in both cases). Participants attributed inadequate knowledge to limited exposure to paediatrics cases, insufficient training, limited scope of practice, and lack of equipment.

**Conclusion:**

Enhancing the knowledge and skills of EMC personnel in paediatrics pre-hospital care through a short learning programme or continuous professional development programme, and providing adequate paediatric emergency equipment, will ensure that comprehensive pre-hospital emergency care is given to paediatric patients in the province.

## Introduction

Accidental life-threatening injuries are among the leading causes of child morbidity (90%) and mortality worldwide [1]. According to a World Health Organization report, injuries and violence account for about 950 000 deaths of children and young people under the age of 18 years each year [1]. Additionally, the childhood injury rate has been reported to be highest in Africa and South Asia [2], with an annual prevalence of 68.2% among 13 to 15 year-olds in six African countries [3]. While prevention is key to reducing death and morbidity caused by life-threatening injuries, providing effective pre-hospital care promptly can, in most cases, curtail the consequences of a life-threatening injury [4]. A coordinated emergency health care system is essential for ensuring proper, effective and timely interventions to prevent mortality [5, 6]. A key component of an emergency health care system is emergency medical care (EMC) personnel who are specialist trained in essential components of a robust acute care system [7].

In South Africa in 2016, injuries accounted for 4,483 deaths of children aged 0-4 years [8]. Since these injuries are often incurred by healthy children who are engaging in daily activities, the injuries can be particularly devastating to the injured child and their families, and may have tragic short or long-term consequences [9]. In a cohort study of critically ill and injured children performed in Cape Town, Hodkinson *et al.* report that delays by the emergency medical services system and poor pre-hospital clinical care are key contributors to the morbidity and mortality of critically ill and injured children [6]. In the Free State province of South Africa, approximately 1 500 EMC personnel are employed across the five districts of the province (Xhariep, Motheo, Fezile Dabi, Lejweleputswa, and Thabo Mofutsanyane). For this present study, we used a questionnaire survey to assess the knowledge of the EMC personnel employed by the Free State Department of Health (DoH) on aspects of paediatric pre-hospital emergency care.

## Methods

This research was designed as a descriptive study that made use of a questionnaire survey.

**Questionnaire survey:** The structured questionnaire used in this study was self-administered and was distributed manually (in hard copy) to the participants during two contact sessions organised for the purpose of this study. The questionnaire was compiled using factors, identified during the literature review, which had been used by previous studies. Some questions were adapted so that they were applicable to the context of the pre-hospital EMC environment. The questionnaire collected data in the following three sections: **Section A:** *Biographical data:* gender, age, race, qualification, district of operation, level of experience, postgraduate qualifications, **Section B:** Knowledge of participants in relation to aspects of paediatrics pre-hospital emergency care, **Section C:** Open-ended questions requested participants to suggest how paediatric pre-hospital emergency care can be improved in the province.

**Assessing the knowledge of EMC personnel on aspects of paediatrics pre-hospital emergency care:** in order to assess participants’ knowledge on aspects of paediatric pre-hospital emergency care, Section B asked participants to provide written answers to seven subject-specific questions relating to aspects of paediatrics emergency care in the pre-hospital setting. The levels of knowledge assessed include, Level 1: Remember (K1) (The ability of participants to recognise, remember and recall terms or concepts); and Level 2: Understand (K2) (The ability of participants to explain ideas or concepts) [10]. The correct answers to the questions used in the questionnaire were sourced from textbooks and national and international standard guides. The correct answers were given to an independent assistant, who marked participants written answers. The independent assistant is a qualified EMC practitioner who is trained in providing pre-hospital emergency care to paediatric patients. One of the researchers, who is a qualified EMC practitioner who has also received training to provide paediatrics pre-hospital emergency care, later crosschecked participants written answers and scores. Participants responses were grouped into correct, incorrect and uncertain (participants did not provide a correct answer, instead, gave incomplete explanations).

**Target population:** The target population was all EMC personnel working in the Free State provincial emergency medical services who were registered (at the time of the study) with the Health Professions Council of South Africa (HPCSA).

**Sample size:** In this study, stratified random sampling was used to obtain a representative sample of 250 EMC personnel working in the Free State provincial emergency medical services. The strata in this study were different levels of employment, as well as the different districts in the Free State province. The survey population consisted of individuals who were willing to sign the consent forms and complete the questionnaire.

**Pilot study:** A pilot study was conducted to test the suitability of the study design and methods, the chosen data collection method and the overall structure of the questionnaire. The pilot study consisted of six EMC personnel at different levels of employment, and in different districts in the province. The findings of the pilot study confirmed the feasibility of the main study, as the participants in the pilot study did not recommend changes to the structured questionnaire. The result of the pilot study was not included in the final results.

**Data collection and analysis:** Data collection was aided by district supervisors in the different regions, who assisted in both the dissemination and collection of the questionnaires. Quantitative data collected from the structured questionnaire was analysed quantitatively and results presented as frequencies and percentages. One-way ANOVA with Newman-Keuls Multiple Comparison Post-Test on Graph Pad Prism 4.0c (Graph Pad, San Diego, CA, USA) was used to determine significant differences between calculated mean percentages. The open-ended questions were analysed by the researchers by reading, identifying and summarising concepts, and grouping themes into specific categories.

**Ethical considerations:** Approval to conduct the research project was obtained from the Health Sciences Research Ethics Committee of the Faculty of Health Sciences at the University of the Free State (Ref. No. ECUFS212/2013). Permission was also obtained from the Free State DoH.

## Results

Only 197 of the initial 250 questionnaires that had been distributed were returned, giving a response rate of 78.8%.

**Demographic information of questionnaire survey participants**

**Age of participants:** The majority of the participants (56.0%) were between 31 and 40 years, 23% were between 41 and 50 years, 13% were aged 26 - 30 years, 3% of the participants were younger than 25 years, and 3% were above 50 years. This data indicates the diversity of participants in relation to the age of EMC personnel in the province. Four (2%) participants did not indicate their ages.

**Gender of participants:** Male participants made up 65% of the group, and female participants 34.5%, which indicates a male predominance in the profession. One (0.5%) of the 197 participants did not indicate a gender.

**Figure 1:**
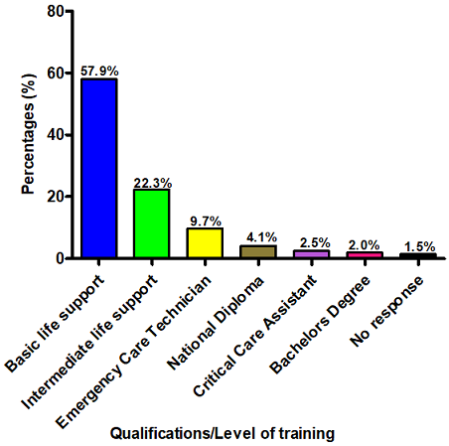
Qualifications/level of training of participants (%) (n = 194)

**Qualification/level of training of participants:** More than half (57.9%; n = 114) the participants had basic life support qualifications, 22.3% (n = 44) had intermediate life support qualifications, and 9.7% (n = 19) were qualified emergency care technicians (Figure 1).

**Desire to obtain further qualification/training in EMC:** The majority (92%) of participants indicated a desire to obtain further qualifications/training in pre-hospital emergency care, while 3% said they did not. Of the participants, 5% did not answer the question (n = 187). The reasons given by those who desire to obtain further qualifications/training in EMC are summarised and presented as themes and categories in Table 1.

**Table 1:** Participants’ reasons for wishing to obtain further qualifications in EMC; n = 197

Themes	Categories
Qualification	Acquire qualification
	Acquire advanced training
Knowledge	Improve knowledge
	Obtain knowledge and skill
	Gain more experience
Patient Care	Improve patient care
	Ensure professional competency
Departmental improvement	Enforce departmental change
	Maintain and keep high standards
	Maintain a high level of professionalism
Staff	Shortage of staff
	Lack of current emergency care technicians
Personal	Personal development and personal reasons
	Increase income
Better service	Better service to community

**Figure 2:**
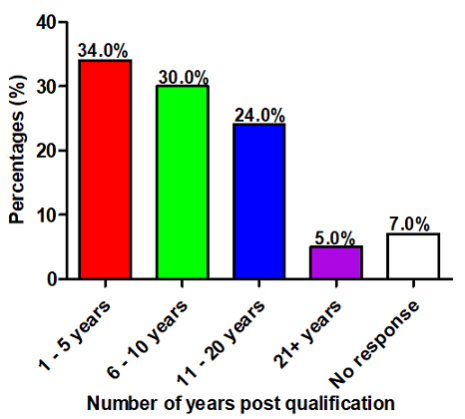
Number of years since the qualifications were obtained (n = 183)

**Number of years post qualification:** The majority (34.0%) of participants had obtained their qualification in the last five years, while only 5.0% had obtained their qualification more than 21 years ago. A further 30.0% and 24.0% of the participants are 6-10 and 11-20 years post qualification, respectively. Seven percent (7%) did not provide information in response to the question (Figure 2).

**Figure 3:**
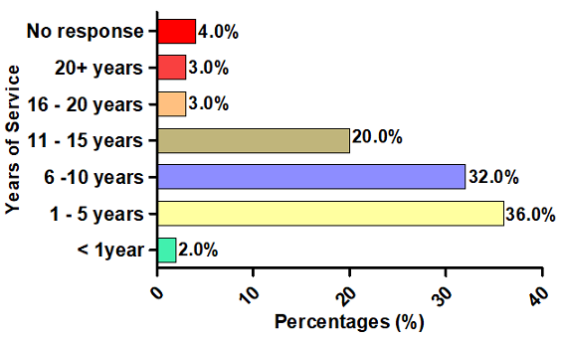
Duration of service as a pre-hospital emergency medical care practitioner (n = 188)

**Duration of service:** The number of years that participants had been working as pre-hospital emergency medical care personnel is presented in **Figure 3**. The majority, that is, 36.0% of participants, indicated that they had been in service for between one and five years. A further 32.0% had worked for 6-10 years, while only 2.0% had less than a year of service (Figure 3). Nine participants (4%) indicated “No response”.

**Location of workplace:** More than half (57.0%) the participants worked in rural settings; the remaining 43.0% worked in urban settings.

**Number of paediatric calls responded to per week:** Participants were asked to indicate the number of paediatrics calls they respond to per week, on average. Most (31.2%) of the participants in the five districts indicated that they respond to an average of 1 paediatrics call per week, while only 13.8% indicated that they respond to an average of 5 or more paediatrics calls (Table 2).

**Table 2:** Average number of paediatric calls participants respond to per week, n = 182

Number of paediatrics calls per week	Participants (%)
0	20.2
1	31.2
2	14.4
3	10.6
4	9.8
≥ 5	13.8

**Knowledge of EMC personnel on aspects of paediatrics pre-hospital emergency care:** This section of the questionnaire focused on assessing participants’ knowledge of aspects of paediatric pre-hospital emergency care.

**Participants self-appraisal of their knowledge of paediatric pre-hospital emergency care:** First, participants were asked to do a self-appraisal of their knowledge of paediatric pre-hospital emergency care by answering Yes or No to the question, Is your knowledge of paediatric pre-hospital emergency care adequate and up to standards? Only 178 participants answered this question. Results reveal that 51.1% (n = 91) of participants across the districts admitted to possessing inadequate knowledge, by answering No.

**Reasons for adequate or inadequate knowledge:** Furthermore, participants were asked to state reasons for believing that their knowledge of paediatric pre-hospital emergency care was adequate or inadequate. While some participants indicated that their knowledge was adequate because of the training they had undergone (cf. quotes #1 - #7), others indicated that lack of exposure, insufficient training, limited scope of practice and lack of equipment were responsible for their inadequate knowledge (cf. quotes #8 - #18). *#1 “I was trained to care and transport this kinds of patient and I attended the PALS training”, #2 “I did a module in paediatric care and transportation”, #3 “Because even now I am still in training”, #4 “Because I am currently busy with my obstetrics classes”, #5 “Due to constant in-service training programmes run in the district”, #6 “Workshops regarding paediatrics were rendered”, #7 “We are working with so many paediatrics, I gained experience”, #8 “Because I've never been exposed to paediatric cases most of the time”, #9 “Need more training on paediatrics”, #10 “People don't know how to treat paediatric-only take temperature”, #11 “I do not have enough training on paediatric patients”, #12 “I am not trained”, #13 “Limited scope of practice”, #14 “My qualification does not allow me to treat paediatric”, #15 “I don't get enough education and training for paediatric prehospital care”, #16 “Because I can only treat to a certain point-it is my protocol”, #17 “Because we don't have equipment for paediatric on the ambulance”, #18 “Because of some lack of information”*

**Participants knowledge on aspects of paediatrics pre-hospital emergency care:** Table 3 shows the percentages of participants responses to questions testing their knowledge, namely, correct, incorrect or uncertain. It is clear that the majority of the participants (53.4% and 74.0% respectively) did not know the scoring of the paediatrics Glasgow Coma Scale (GCS), and were unable calculate paediatric blood pressure (Q5 and Q1, P<0.0001 for both cases). More participants knew how to perform cardio-pulmonary resuscitation (CPR) (61.4%; P < 0.0001), use the resuscitation/ Broselow tape, and determine capillary refill time for infants (Table 3).

**Table 3:** Knowledge of EMC personnel on aspects of paediatrics pre-hospital emergency care

Questions	Participants’ answers		
	Correct (%)	Incorrect (%)	Uncertain (%)
Question 1: Write down the formula to calculate paediatric blood pressure (n = 117)	3.4	74.0***	22.6
Question 2: Write down the steps in cardio pulmonary resuscitation (CPR) as stated in the guideline for paediatric patients (n = 168)	61.4***	28.0	10.6
Question 3: Write down the steps in the paediatrics choking treatment algorithm (n = 124)	46.2	35.0	18.8
Question 4: Write down the steps in foreign body airways obstruction (FBAO) treatment on an unresponsive paediatric (n = 153)	28.2	39.8	32.0
Question 5: Write down the scoring for paediatric Glasgow Coma Scale (n = 120)	20.0	53.4***	26.6
Question 6: Write down how to use the resuscitation/ Broselow tape (n =69)	53.8	41.6	4.6
Question 7: Write down steps to determine capillary refill time in an infant (n = 143)	51.6	43.8	4.6

*** P < 0.0001

**Improving paediatric pre-hospital emergency medical care in the Free State province:** This section explored participants opinions on how emergency medical care in the Free State province could be improved. The suggestions given by the participants were categorised into themes, and some of the relevant themes and their weights (total number of votes received) are presented in Table 4. More than half (n=103) of the participants proposed In-service training and education, and 41 participants suggested that providing the necessary equipment and consumables, and increasing the number of specially equipped vehicles to treat and transport paediatrics would improve paediatrics care, while others (n=33) said staff should be made to attend training courses (Table 4). Other suggestions proposed by the participants include increasing staff (n=8), increasing the advance trained personnel (n=8), improving management (n=3), and receiving exposure in paediatric units (n=1) (Table 4).

**Table 4:** Participants’ suggestions for improving paediatric pre-hospital emergency medical care in the Free State province; n = 178

Themes	Total number of votes (n)
In-service training and education	103
Provide the necessary equipment and consumables to treat paediatrics	41
Increase number of specially equipped vehicles for paediatric transportation	41
Attend training courses	33
Increase staff	8
Increase the advance trained personnel	8
Better management	3
Improve personnel attitude/morale and motivation	3
Sending qualified personnel while transporting patients	3
Educating the community about EMS services	2
Retain qualified personnel who move to private sector	2
Roadworthy vehicles	2
Senior management should address the issues	1
Exposure to paediatric units	1
Implementation of quality assurance strategies to keep parties accountable	1
Staff development for better service delivery	1
Invest in quality personnel by sending them for M.Tech and PhD	1
Cut out delays when attending paediatric cases	1

## Discussion

Pre-hospital emergency medical personnel respond to and manage diverse urgent medical situations, including paediatrics emergencies. Children account for 10% of EMC incidents [11] and their pre-hospital emergency needs differ greatly from that of adult patients [12]. Noteworthy is that the pattern of paediatrics emergencies may vary regarding location (rural or urban) [13], gender, and age category [14], season of the year, day of the week, and time of call [15]. According to the Institute of Medicine Committee on Paediatric Emergency Medical Services report [16], curricula for pre-hospital care providers should cover the emergency care of children, including resuscitation [16]. However, most training programmes concentrate on cardiac life support for adults, and even the most comprehensive programmes devote only three or four lectures to paediatric emergencies [12]. Many EMC units do not have appropriate paediatric resuscitation equipment and medications, making it difficult, if not impossible, to provide adequate emergency care for critically ill children [12]. A survey conducted by Houston *et al.* (2010) reports that paediatrics pre-hospital emergency care by frontline personnel (paramedics) in the United Kingdom is limited by lack of resources, and by staff with only basic skills [17]. Similarly, in Cape Town, South Africa, the poor quality of pre-hospital emergency care that results from poor or limited personnel training has been identified as a key contributor to the morbidity and mortality of critically ill and injured children [6, 18].

Historically, the pre-hospital emergency medical care environment has been a male-dominated field [19], and concerns have been raised about gender bias in the profession [20]. The distribution of 65% men and 35% women found by this study confirms published literature [20-22] and, thus, suggests that the Free State EMC service is dominated by male practitioners. This dominance is can be explained partly by the fact that, prior to the establishment of EMC as a profession, pre-hospital emergency medical services had been offered by staff of fire departments, who were, in most communities, including the Free State, traditionally men [19, 20]. Data obtained by this study suggests that the majority of the EMC personnel employed by the Free State DoH are in possession of only basic or intermediate life support certification (Figure 1). The Basic Life Support (BLS) certification is a 4-6 week course that offers training in only basic life support (including both CPR and first aid) and the use of ambulance equipment [23]. Staff who have undergone basic ambulance attendant (BAA) training can provide basic treatment, including (but not limited to) administering oxygen and splinting fractures [24], but they lack training in paediatrics pre-hospital emergency care. This limitation may have an impact on the scope of pre-hospital emergency care delivered by such personnel and, ultimately, patient outcomes [25]. Prior studies have reported a positive correlation between paramedics years of experience and patient outcomes in the pre-hospital environment [26, 27] *Soo et al.* (1999) report that the chances of survival in an out-of-hospital cardiac arrest (OHCA) case increases when an emergency care technician possesses over five years of experience after qualification, and in the case of paramedics, after just one year of experience [26]. The finding of this study that the majority of participants had between 1 and 10 years experience post qualification suggests that EMC personnel of the Free State DoH have a high probability of achieving patient survival.

Performance appraisal systems are reported to be a vital tool for identifying staff training needs and helping employees meet performance targets [28]. In a self-appraisal of their knowledge of paediatrics pre-hospital emergency care, more than half the participants across the districts (51.2%) reported inadequate knowledge, which they ascribed to limited exposure to paediatrics cases, insufficient training, limited scope of practice and lack of equipment (cf. quotes #8- #18). In a study that investigated whether previous paramedic exposure to OHCA resuscitation is associated with patient survival, Dyson *et al.* (2015) report that patient survival increases significantly with the number of OHCAs that paramedics have treated [29]. This finding suggests, thus, that limited exposure to paediatrics cases, as reported by Free State EMC personnel in this study (Table 2), may lead to poor patient outcomes. A feasible solution to counteract the effect of limited exposure will be using high-fidelity simulation training to improve cognitive performance by EMC personnel [30]. Including basic principles of pre-hospital paediatrics emergency care in the curricula for pre-hospital care providers (including short courses), as proposed by the Institute of Medicine Committee on Paediatric Emergency Medical Services [16], will enhance training. Several studies have documented deficiencies in availability of equipment and supplies appropriate for paediatric patients, on ambulances and in emergency departments [11]. The lack of equipment reported by participants of this study confirms these findings by Anest *et al.* (2016), who, in their Cape Town study, report that lack of paediatric-specific equipment, such as incubators, appropriate blood pressure cuffs and ventilators with paediatric settings, constitute a major barrier to pre-hospital care of paediatric patients [18].

An assessment of participants knowledge of aspects of paediatrics pre-hospital emergency care, revealed that the majority of EMC personnel in the service of the Free State DoH cannot calculate paediatric blood pressure (74.0%; P < 0.0001). Paediatric resuscitation guidelines make it clear that it is important to measure blood pressure when assessing a sick child's circulatory and neurological status [31]. Comparing measured values to a chart or calculated blood pressure for age (in children over 1 year) assists paramedics in diagnosing hypo- or hypertension. Hypotension is a late and pre-terminal sign of circulatory failure. Once a child's blood pressure has fallen, cardiac arrest is imminent; [31] thus, failure by EMC personnel to accurately diagnose hypotension could have dire consequences. Although the majority of the participants were knowledgeable about the new CPR guidelines for paediatric patients, more than half (53.4%; P < 0.0001) of the participants did not know the paediatric GCS (Table 3). The GCS score is the most commonly tool used to assess the severity of traumatic brain injury in both adults and children [32] and has been reported to be the most feasible, accessible and reliable predictor of traumatic brain injury outcome in paediatrics [33]. Inadequate knowledge of the GCS score in participants of this study suggests that EMC personnel in the employ of the Free State DoH cannot accurately assess the severity of traumatic head injury, or the state of impairment on the consciousness continuum [34].

Finally, participants advocated that providing in-service training and education is the major way by which EMC for paediatric patients could be improved in the Free State province (Table 4). Moreover, the majority (92%) of participants indicated a desire to obtain further qualifications/training in pre-hospital emergency care, in order to improve their qualifications and knowledge and to enhance patient care and services (Table 1).

## Conclusion

EMC personnel employed by the Free State DoH are often the first responders at scenes of medical emergency, including paediatrics emergencies. Enhancing their knowledge and skills in paediatrics pre-hospital emergency care will ensure that adequate and comprehensive pre-hospital emergency care is available for paediatrics patients in the Free State province. The researchers propose the following recommendations to enhance the knowledge of EMC personnel in the province on paediatrics pre-hospital emergency care: Create an education platform to train all EMC personnel in the province on the basic principles and practice of paediatrics pre-hospital emergency care; This education platform can be presented as a short learning programme or as continuous professional development, in conjunction with the University of the Free State (Simulation Unit) and the Free State College of Emergency Care.

### What is known about this topic

- It has been established that there is a poorly coordinated emergency health care system in some parts of Africa;
- In Cape Town, South Africa, poor quality of pre-hospital emergency care, which is the result of poor or limited personnel training, has been identified as a key contributor to the morbidity and mortality of critically ill and injured children.

### What this study adds

- This study found that some EMC personnel employed by the Free State DoH are not knowledgeable about aspects of paediatrics pre-hospital emergency care (e.g. calculating paediatric blood pressure for age and scoring for paediatric GCS);
- This study adds, furthermore, that poor quality paediatrics pre-hospital emergency care that is the result of lack of knowledge by EMC personnel could lead to increased morbidity and mortality of critically ill and injured children in the Free State province;
- To address this knowledge gap and prevent deaths, this study offers implementable recommendations (creation of an education platform in the form of a short learning programme or continuous professional development) to enhance the knowledge of EMC personnel in the province on paediatrics pre-hospital emergency care.
